# Safety and Efficacy of Riluzole in Traumatic Spinal Cord Injury: A Systematic Review With Meta-Analyses

**DOI:** 10.1089/neur.2023.0114

**Published:** 2024-02-19

**Authors:** Luke J. Weisbrod, Thomas T. Nilles-Melchert, Judith R. Bergjord, Daniel L. Surdell

**Affiliations:** ^1^ Department of Neurosurgery, University of Nebraska Medical Center, Omaha, Nebraska, USA.; ^2^ School of Medicine, Creighton University, Omaha, Nebraska, USA.; ^3^ Health Sciences Library, Creighton University, Omaha, Nebraska, USA.

**Keywords:** riluzole, spinal cord injury, trauma

## Abstract

Traumatic spinal cord injury (SCI) is a cause of significant morbidity, often resulting in long-term disability. We aimed to compare outcomes after riluzole versus patients who received placebo or standard of care with no specific intervention. MEDLINE, Embase, Scopus, and Cochrane Library database searches yielded 92 records, and five met the study inclusion criteria. Fixed-effect and random-effects models were used to establish odds ratios (ORs) and mean difference (MD) with 95% confidence intervals (CIs) for each outcome. The results of the pooled analysis showed that in patients with acute traumatic SCI, riluzole resulted in increased American Spinal Injury Association (ASIA) motor scores at 3 months (MD 0.26, 95% CI [–0.10,0.61], *I^2^* = 0%; *p* = 0.157) and 6 months (MD 0.21, 95% CI [–0.17,0.60], *I*^2^ = 0%; *p* = 0.280) and change in ASIA Impairment Scale (AIS) at 3 months (OR 0.59, 95% CI [–0.12,1.30], *I*^2^ = 0%, *p* = 0.101) and 6 months (OR 0.28, 95% CI [–0.50,1.06], *I*^2^ = 0%, *p* = 0.479) in comparison to the control groups, though not to a level of statistical significance. Riluzole resulted in fewer adverse events than the control groups (OR −0.12, 95% CI [–1.59,1.35], *I*^2^ = 0%, *p* = 0.874) and lower mortality (OR −0.20, 95% CI [–1.03,0.63], *I*^2^ = 0%, *p* = 0.640), though also not to a level of statistical significance. These meta-analyses suggest that riluzole for the treatment of traumatic SCI is safe and results in improved neurological outcomes when compared to controls, though not to a level of statistical significance. More robust prospective, randomized studies are necessary to help inform the safety and efficacy of riluzole for traumatic SCI.

## Introduction

The National Spinal Cord Injury Statistical Center has estimated the annual incidence of spinal cord injury (SCI) in the United States at ∼17,730 new cases annually, with ∼54 cases per million population.^1^ The prevalence of SCI in the United States has been estimated to be ∼291,000, or around ∼881 per million population.^1,2^ The mean age at the time of injury is 33 years.^3^ SCI is accompanied by vast, prolonged social, physical, and monetary costs to persons and society, including the initial hospitalization, acute rehabilitation post-discharge, modifications at home and to motor vehicles, and recurring costs for medications, durable medical equipment, supplies, and personal assistance. The extent of disability varies depending on the severity, level of injury, and whether additional injuries were present, as is common for polytrauma. On average, it has been estimated that a person with SCI will incur $1,130,000 in direct costs in the first year after the accident, followed by $196,107 annually thereafter.^4^

The pathophysiology of SCI is comprised of two sequential processes: primary and secondary injuries. Primary injury is the mechanical injury resulting from the trauma itself and can only be treated by accident prevention. Secondary injury is characterized by a cascade of biological reactions triggered by the primary injury and includes ischemia, hemorrhage, inflammation, oxidative stress, and apoptotic pathways, among others.^5^ The secondary injury is therefore the primary target for the treatment of SCI and has been the focus of extensive research. By therapeutically attenuating the secondary injury, the spread of spinal cord parenchymal damage is suppressed, with the goal of improved neurological and functional prognoses.^6^

The acute management of SCI includes clinical assessment, characterization of the injury, immobilization with strict spinal precautions until instability has been excluded, and critical care monitoring of hemodynamics and respiratory status with maintenance of mean arterial pressure >85 mm Hg in the first 5–7 days post-injury and early surgical decompression and/or stabilization in appropriate cases.^7^ Subsequent to immediate stabilization, measures are taken to prevent complications secondary to immobility, including deep venous thromboses, pneumonia, and pressure ulcers, among others. Rehabilitation during inpatient admission and post-discharge is essential to optimizing function in patients with SCI.^8^

Aside from the above core measures, there has not been a pharmacological therapeutic agent that has been shown to safely and effectively improve outcomes in patients who have suffered from traumatic SCI. Historically, a treatment option was the early administration of high-dose methylprednisolone (MP). The mechanism of action of MP was to prevent secondary injury through anti-inflammatory effects and stabilization of the cell membrane.^9^ In the second National Acute Spinal Cord Injury Study (NASCIS-2) in 1990, a small beneficial effect of high-dose MP was reported for neurological recovery if administered within 8 h post-injury in patients with traumatic SCI.^9^ This conclusion, however, was drawn from *post hoc* subgroup analysis, which challenged the level of evidence.^10^ Recently, the inefficacy and significant potential for adverse effects and even death have been reported.^11,12^ As a result, the majority of up-to-date guidelines on the pharmacological therapy for acute SCI do not recommend steroids and its use is controversial.^13,14^ There is therefore a significant need for a novel drug that can attenuate secondary injury post-SCI without substantial associated adverse effects.

Riluzole is a sodium channel-blocking benzothiazole anticonvulsant that exerts neuroprotective effects by reducing the release of excitotoxic glutamate and helping to maintain neuronal cellular ionic balance.^15^ Riluzole is approved by the U.S. Food and Drug Administration (FDA) for the chronic treatment of patients with amyotrophic lateral sclerosis (ALS), a progressive neurodegenerative disorder characterized by motor neuron and corticospinal tract degeneration.^16^

There is evidence from pre-clinical traumatic SCI animal models that riluzole attenuates the secondary injury cascade that leads to neurological tissue destruction.^17,18^ After traumatic SCI, there is an increase in glutamate release and cell membrane permeability, which results in prolonged excitability of post-synaptic neurons, ultimately leading to neuronal edema and cell death.^17^ Through inhibition of voltage-gated sodium channels, riluzole functions to inhibit glutamate release and neuronal excitotoxicity.^17^ Over the past decade, the use of riluzole in traumatic SCI has been translated clinically into prospective studies. The purpose of this systematic review with meta-analyses was to determine the effect of riluzole in patients with traumatic SCI on neurological and functional outcomes, as well as its safety with respect to adverse events and mortality.

## Methods

### Search strategy

A comprehensive, systemic literature search was conducted through the Cochrane Library, Embase, MEDLINE, and Scopus databases on October 30, 2023, for articles investigating the safety and efficacy of riluzole compared to placebo or standard management of traumatic SCI following PRISMA (Preferred Reporting Items for Systematic Reviews and Meta-Analyses) guidelines.^19^ The search strategies included the title and keywords that included the two search concepts: 1) riluzole and 2) spinal cord injury. We focused the search on work with adult patients by first removing articles indexed as concerning animals if these were not also indexed as concerning humans and then removing articles indexed as concerning pediatric age groups if these were not also indexed as concerning adults. Because funds were not available for translation, searches were limited to English-language articles, and because full study data were needed, conference abstracts, book chapters, and clinical trial registry records were excluded. The search yielded a total of 92 titles/abstracts for review.

Two independent researchers (L.W. and T.N.) screened the 92 articles identified by the search strategy. Seventy-two articles were excluded during the initial screening and 20 articles were assessed for eligibility. Of these 20 articles, five were included for analysis and 15 were excluded. Reasons for exclusion included duplicated patient population (eight) and lack of pre-specified data (seven).

### Selection criteria

We included all English-language articles that evaluated the safety and efficacy administration of the administration of riluzole for the treatment of traumatic SCI in comparison to placebo or supportive care without specific additional intervention. Criteria for inclusion in the study were: 1) traumatic etiology of SCI; 2) a sample size of ≥5 patients in each group; 3) adult patient population with age ≥18 years; and 4) available data regarding American Spinal Injury Association (ASIA) motor score, change in ASIA Impairment Scale (AIS), ASIA sensory recovery, functional outcome with Spinal Cord Independence Measure (SCIM) version III, adverse events, and mortality. Studies with overlapping patient data already included for the analysis and lack of pre-specified data were excluded.

### Outcomes

The outcomes of interest in this study included changes to AIS (defined as an increase in at least one grade), ASIA motor score, ASIA sensory score, SCIM III scores, adverse events, and mortality.

### Data extraction

Data were extracted independently by two researchers (L.W. and T.N.) and were collected using Microsoft Excel (Microsoft Corp., Redmond, WA). We recorded the following information: last name of the first author and year of study, location in which the study occurred, study dates, number of patients included in the study, mean age, sex ratio, riluzole dose, control group intervention, acuity of SCI, spinal cord level of injury, severity of SCI, if surgery was performed, changes to AIS (defined as an increase in at least one grade), ASIA motor score and ASIA sensory scores, SCIM III scores, adverse events, and mortality.

### Quality assessment

The Newcastle-Ottawa Scale was used to assess the quality of included studies that did not have a prospective, randomized design.^20^ Two reviewers (L.W. and T.N.) performed the quality assessments individually, and any discrepancies were resolved with discussion. Studies rated with zero to three stars were considered low quality, studies with four to six stars were considered medium quality, and studies with seven to nine stars were considered high quality.

### Statistical analysis

Meta-analyses were performed on outcomes of interest if three or more study populations were available for pooled analysis of designated outcomes of interest. Meta-analyses were performed to calculate pooled odds ratios (ORs) and mean difference (MD) with 95% confidence intervals (CIs) using a fixed-effect model for variables with low heterogeneity as measured by the *I*^2^ statistic and a random-effects model for continuous variables with higher risk for heterogeneity as measured by the *I*^2^ statistic. *I*^2^ values <25% were considered to have low heterogeneity, whereas all others were considered to have higher heterogeneity and were analyzed using the random-effects model. Statistical significance was achieved with a *p* value <0.05. Results are presented in forest plots. All analyses were completed using the meta-analysis functions in the open statistical software Jamovi.^21^

## Results

### Search results

**FIG. 1.**
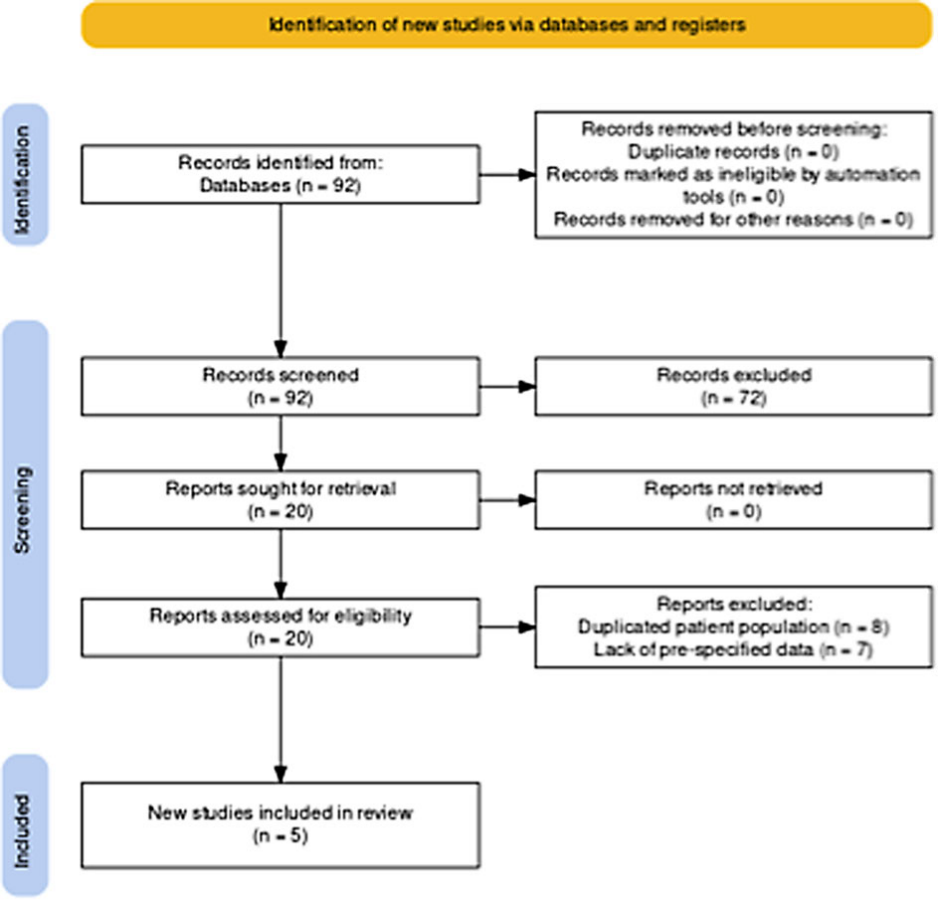
PRISMA (Preferred Reporting Items for Systematic Reviews and Meta-Analyses flowchart).

MEDLINE, Embase, Scopus, and Cochrane Library databases identified 92 publications (Fig. 1). In the final meta-analyses, five studies were included after studies that failed to meet inclusion criteria were removed. The characteristics of the five studies included in the meta-analyses are presented in Table 1.^22–26^ Two studies were performed across North America, one study was performed across North America and Australia, one study was performed in India, and one study was performed in Iran. Four studies were prospective and randomized, and one study was prospective and non-randomized. The control intervention was standard of care without specific intervention in three studies and placebo in two studies. The dose and duration of riluzole varied across studies and included 100 mg twice-daily (BID) for 1 day followed by 50 mg BID for 13 days in four studies and 50 mg BID for 8 weeks in one study. Regarding acuity of injury, all studies were within the acute period post-SCI. In two studies, administration of riluzole occurred within 12 h of injury, in one study administration of riluzole occurred within 72 h of injury, in one study administration of riluzole occurred within 96 h of injury, and in one study the precise timing was not specified more than to say that it was within the acute period post-injury.

**Table 1.** Characteristic of Studies Included in Meta-Analyses

***Study***	***Study location***	***Study design***	***Study dates***	***Newcastle-Ottawa Score***	***Control***	***Dose riluzole***	***Acuity***
**Kumarasamy et al.^23^**	India	PR	April 2018 to March 2019	NA	SC	100 mg BID then 50 mg BID 13 days	<72 h
**Fehlings et al.^22^**	North America, Australia	PR	October 2013 to May 2020	NA	P	100 mg BID then 50 mg BID 13 days	<12 h
**Grossman et al.^24^**	North America	PNR	April 2010 to June 2011	8	SC	100 mg BID then 50 mg BID 13 days	<12 h
**Chow et al.^25^**	North America	PR	October 2013 to May 2020	NA	P	100 mg BID then 50 mg BID 13 days	<96 h
**Meshkini et al.^26^**	Iran	PR	2014 to 2015	NA	SC	50 mg BID 8 weeks	Acute

PR, prospective randomized; PNR, prospective non-randomized; NA, not applicable; P, placebo; SC, standard of care; BID, twice a day.

The meta-analyses included 378 patients whose demographics are included in Table 2. Of the 378 total patients, 187 received riluzole. The control groups included a total of 191 patients; 115 patients received a placebo and 76 patients received standard of care without a specific placebo intervention. The range of the mean age of the riluzole and control groups was 37.7–51.4 and 36.9–51.3, respectively. The mean age was not specified for the riluzole or control group in one study. An elevated male-to-female ratio was observed in all riluzole and control groups. The range of male to female ratios of the riluzole and control groups ranged from 1.70 to 4.65 and 1.7 to 6.2, respectively. The male-to-female sex ratio was not specified for the riluzole or control group in one study.

**Table 2.** Characteristics of Patient Demographics Included in Meta-Analyses

***Study***	***N R***	***N Cl***	***Mean age R***	***Mean age Cl***	***Sex ratio M:F R***	***Sex ratio M:F Cl***	***Level***	***Severity***	***Surgery?***
Kumarasamy et al.^23^	23	20	47.7	51.2	NA	NA	C	I	Yes
Fehlings et al.^22^	96	97	49.4	47.6	4.65	4.39	C	I/Co	Yes
Grossman et al.^24^	24	26	NA	NA	5	6.2	C/T	I/Co	Yes
Chow et al.^25^	14	18	51.4	51.3	3.7	3.5	C	I/Co	Yes
Meshkini et al.^26^	30	30	37.7	36.9	1.7	1.7	C/T/L	I/Co	NA

N, number; R, riluzole; Cl, control; M, male; F, female; C, cervical; T, thoracic; L, lumbar; I, incomplete spinal cord injury; Co, complete spinal cord injury; NA, not available.

In three studies, the level of SCI was cervical spine only. In one study, the level of SCI included the cervical and thoracic spinal cord, and in one study the level of SCI included the cervical, thoracic, and lumbar spine. In four studies, the severity of SCI included complete and incomplete SCI. In one study, the severity of SCI included only patients with incomplete SCI. Four studies commented that surgical decompression and/or fixation was performed if indicated based on surgeon discretion during a therapeutic window that they deemed acceptable. The four studies that commented on surgical intervention performed early surgery <24 h from the time of injury and once stabilized. The remaining one study did not mention whether surgical intervention was performed.

The adverse events and mortality of patients included in the meta-analyses are included in Table 3. There were 13 reported mortalities in the riluzole group and 15 reported mortalities in the control group. There were a total of 1781 adverse events reported of the 187 patients who received riluzole. There were no reported adverse events that were directly attributable to riluzole. Specific reported adverse events in the riluzole group included infection (14), pulmonary (11), neuropsychiatric (10), hematological (seven), cardiovascular (five), gastrointestinal/genitourinary (GI/GU; five), skin (four), wound infection (one), atrial fibrillation (one), and durotomy with a cerebrospinal fluid leak (one). In the control groups, there was a total of 1858 adverse events reported of the 191 patients. Specific reported adverse events in the control group included pulmonary (16), infection (13), cardiovascular (11), GI/GU (nine), neuropsychiatric (eight), skin (three), respiratory distress (one), aspiration (one), and hematoma (one).

**Table 3.** Adverse Events and Mortality of Patients Included in Meta-Analyses

	***R 50 mg BID 8 weeks***	***R 100 mg BID then 50 mg BID 13 days***	***R total***	***Control***	***Total***
Patients N	30	157	187	191	378
Patients w/ any AE	NA	1781	1781	1858	3639
AE related to riluzole	NA	0	0	—	0
Mortality	NA	13	13	15	28
Durotomy w/ CSF leak		1	1		1
Atrial fibrillation		1	1		1
Wound infection		1	1		1
Infection		14	14	13	27
Respiratory distress				1	1
Pulmonary		11	11	16	27
Neuropsychiatric		10	10	8	18
Skin		4	4	3	7
Hematological		7	7	9	16
Cardiovascular		5	5	11	16
GI/GU		5	5	9	14
Aspiration				1	1
Hematoma				1	1

N, number; AE, adverse events; w/, with; CSF, cerebrospinal fluid; GI, gastrointestinal; GU, genitourinary.

### Meta-analyses

There was a sufficient number of study populations to perform meta-analyses on ASIA motor scores at baseline, 3 months, and 6 months post-injury, as well as AIS at 3 months and 6 months post-injury, adverse events, and mortality. There was an insufficient number of study populations to perform meta-analyses on ASIA sensory scores and functional outcomes with SCIM III scoring.

#### American Spinal Injury Association Impairment Scale 3 months

**FIG. 2.**
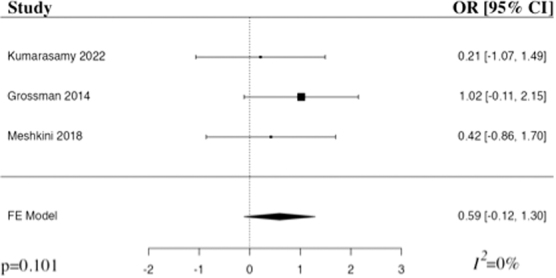
Forest plot demonstrating fixed-effects model for change in AIS at 3 months in patients who received riluzole in comparison to control for traumatic SCI.^22,24,26^ OR, odds ratio; CI, confidence interval; FE, fixed-effects.

The meta-analysis of change in AIS at 3 months included three studies.^22,24,26^ The results of the pooled analysis showed that the administration of riluzole resulted in an increased change in AIS at 3 months in comparison to the control in patients with traumatic SCI, but not to a level of statistical significance (OR 0.59, 95% CI [–0.12,1.30], *I*^2^ = 0%, *p* = 0.101; Fig. 2). The results remained statistically insignificant when additionally run with a random-effects model.

#### American Spinal Injury Association Impairment Scale 6 months

**FIG. 3.**
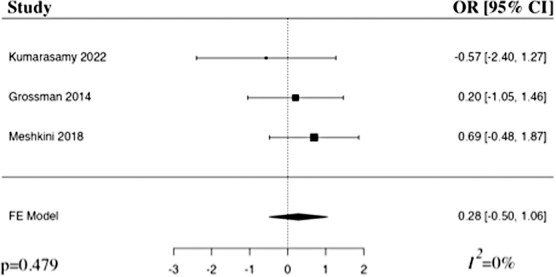
Forest plot demonstrating fixed-effect model for change in AIS at 6 months in patients who received riluzole in comparison to control for traumatic SCI.^22,24,26^ OR, odds ratio; CI, confidence interval; FE, fixed-effects.

The meta-analysis of change in AIS at 6 months included three studies.^22,24,26^ The results of the pooled analysis showed that the administration of riluzole resulted in an increased change in AIS at 6 months in comparison to the control in patients with traumatic SCI, but not to a level of statistical significance (OR 0.28, 95% CI [–0.50,1.06], *I*^2^ = 0%, *p* = 0.479; Fig. 3). The results remained statistically insignificant when additionally run with a random-effects model.

#### American Spinal Injury Association motor baseline

**FIG. 4.**
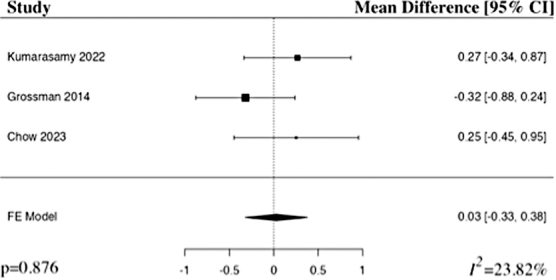
Forest plot demonstrating fixed-effect models for MD of baseline ASIA motor scores in patients with traumatic SCI who received riluzole in comparison to control.^22,24,25^ CI, confidence interval; FE, fixed-effects.

The meta-analysis of ASIA motor baseline included three studies.^22,24,25^ The results of the pooled analysis showed there was no statistically significant difference in ASIA motor baseline in the riluzole group in comparison to the control group in patients with acute traumatic SCI (MD 0.03, 95% CI [–0.33,0.38], *I*^2^ = 23.82%, *p* = 0.876; Fig. 4). The results remained statistically insignificant when additionally run with a random-effects model.

#### American Spinal Injury Association motor 3 months

**FIG. 5.**
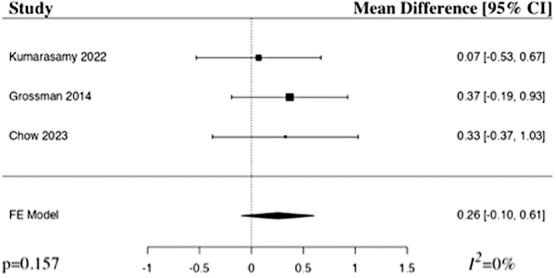
Forest plot demonstrating fixed-effects model for MD in ASIA motor score at 3 months in patients with traumatic SCI who received riluzole in comparison to control.^22,24,25^ CI, confidence interval; FE, fixed-effects.

The meta-analysis of ASIA motor score at 3 months included three studies.^22,24,25^ The results of the pooled analysis showed that the administration of riluzole resulted in an increase in the ASIA motor score at 3 months in comparison to control in patients with acute traumatic SCI, but not to a level of statistical significance (MD 0.26, 95% CI [–0.10,0.61], *I^2^* = 0%, *p* = 0.157; Fig. 5). The results remained statistically insignificant when additionally run with a random-effects model.

#### American Spinal Injury Association motor 6 months

**FIG. 6.**
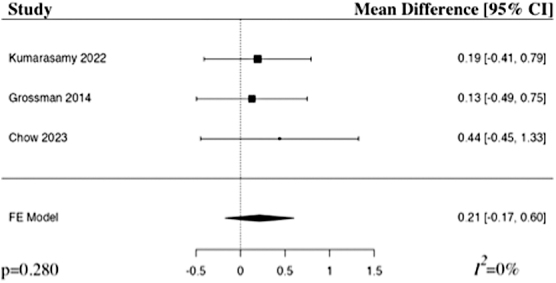
Forest plot demonstrating fixed-effects model for MD in ASIA motor score at 6 months in patients with traumatic SCI who received riluzole in comparison to control.^22,24,25^ CI, confidence interval; FE, fixed-effects.

The meta-analysis of ASIA motor score at 6 months included three studies.^22,24,25^ The results of the pooled analysis showed that the administration of riluzole resulted in an increase in the ASIA motor score at 6 months in comparison to control in patients with acute traumatic SCI, but not to a level of statistical significance (MD 0.21, 95% CI [–0.17,0.60], *I^2^* = 0%, *p* = 0.280; Fig. 6). The results remained statistically insignificant when additionally run with a random-effects model.

#### Adverse events

**FIG. 7.**
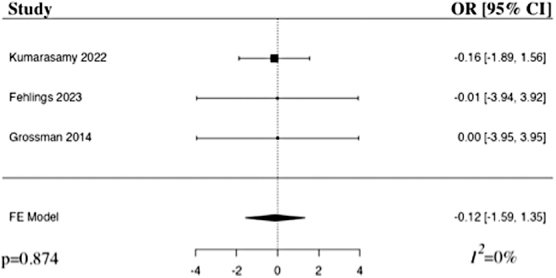
Forest plot demonstrating fixed-effects model for adverse events in patients who received riluzole in comparison to control for traumatic SCI.^22–24^ OR, odds ratio; CI, confidence interval; FE, fixed-effects.

The meta-analysis of adverse events after the administration of riluzole in comparison to control included three studies.^22–24^ The results of the pooled analysis showed that the administration of riluzole resulted in a decrease in adverse events in comparison to control in patients with acute traumatic SCI, but not to a level of statistical significance (OR −0.12, 95% CI [–1.59,1.35], *I*^2^ = 0%, *p* = 0.874; Fig. 7). The results remained statistically insignificant when additionally run with a random-effects model.

#### Mortality

**FIG. 8.**
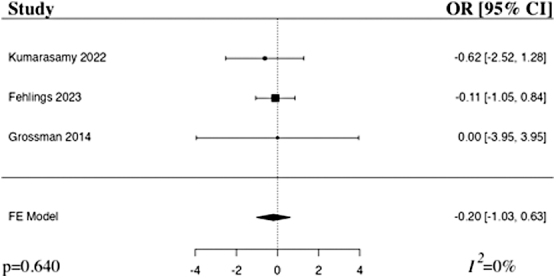
Forest plot demonstrating fixed-effects model for mortality in patients who received riluzole in comparison to control for traumatic SCI.^22–24^ OR, odds ratio; CI, confidence interval; FE, fixed-effects.

The meta-analysis of mortality after the administration of riluzole in comparison to control included three studies.^22–24^ The results of the pooled analysis showed that the administration of riluzole resulted in a decrease in mortality in comparison to control in patients with acute traumatic SCI, but not to a level of statistical significance (OR −0.20, 95% CI [–1.03,0.63], *I*^2^ = 0%, *p* = 0.640; Fig. 8). The results remained statistically insignificant when additionally run with a random-effects model.

## Discussion

Traumatic SCIs are devastating injuries. Secondary injury in SCI is characterized by a cascade of biological reactions triggered by the primary injury and initial insult.^5^ The secondary injury is the primary target for the treatment of SCI and has been the focus of extensive research. Aside from supportive care and early surgical decompression with instrumented fusion when indicated, there has not been a pharmacological therapeutic agent that has been shown to safely and effectively improve outcomes in patients who have suffered from traumatic SCI.^27^

Riluzole is a sodium channel blocker that is currently approved by the FDA for the chronic treatment of patients with ALS, a progressive neurodegenerative disorder.^16^ Given its potential neuroprotective properties, its potential use has been expanded and investigated in the setting of cervical myelopathy, brachial plexus avulsion injuries, and cervical root injuries.^28–30^ Through inhibition of voltage-gated sodium channels, riluzole functions to inhibit glutamate release and neuronal excitotoxicity, effects that are fundamental to the secondary cascade in SCI.^17^ Given riluzole's promise in pre-clinical SCI animal models, its use has been translated clinically into clinical studies. The role of riluzole in the setting of traumatic SCI remains unclear. This systematic review with meta-analyses was planned to further evaluate the safety and efficacy of riluzole through analysis of available pooled data from patients with acute traumatic SCI.

The results of our meta-analyses show that riluzole is a reasonably safe medication in the setting of acute SCI, given that there was no statistically significant increase in adverse events or mortalities in the riluzole group in comparison to the control groups. Riluzole is not associated with any potent cardio- or neurotoxic adverse effects, though elevation of liver enzymes has been reported in patients undergoing treatment with riluzole for ALS.^31^ Elevation of liver enzymes has also been reported to occur acutely in animal models and patients with SCI, possibly as a result of impaired liver perfusion.^32–34^ The effects of liver enzymes from both SCI itself and riluzole therapy were investigated by Grossman and colleagues.^24^ Their study confirmed that mild-to-moderate elevations of aspartate aminotransferase (AST) and alanine aminotransferase (ALT) was observed within the first day of SCI before administration of riluzole in up to 37% of patients, consistent with initial reports by Shepard and Bracken.^24,32^ Grossman also reported a transient, mild-to-moderate elevation of AST, ALT, gamma-glutamyl transferase, alkaline phosphatase, and bilirubin, with up to 70% of patients experiencing an elevation in at least one enzyme.^24^ The bilirubin level had normalized on the last day of riluzole administration on day 14, and the enzyme elevations had normalized by 3–6 months post-injury. There were no reported serious adverse events related to the transient elevations of liver enzymes.

The results of our meta-analyses show a possible modest benefit of riluzole in comparison to controls in neurological outcomes with respect to ASIA motor scores at 3 and 6 months post-injury, as well as change in AIS at 3 and 6 months post-injury, though not to a level of statistical significance. The results of our meta-analyses for change in AIS and ASIA motor outcomes at 6 months were consistent with the findings from Fehlings and colleagues “Safety and Efficacy of Riluzole in Acute Spinal Cord Injury Study” (RISCIS) trial, the largest riluzole patient population to date whose neurological outcomes data were not immediately available for inclusion in our meta-analyses.^23^ The associated complications and mortality provided from the trial were available and included in our meta-analyses. Additionally, results from a substudy from the RISCIS trial by Chow and colleagues were available and included in our meta-analyses for ASIA motor scores.^25^ The RISCIS study was powered at a planned enrollment of 351 patients, but had to be terminated prematurely by the sponsor because of the COVID-19 pandemic. The study was still able to include 96 patients in the riluzole group and 97 patients in the placebo control group and found that, at 6 months post-injury, there was an increase in ASIA motor scores (*p* = 0.3490) and change in AIS by one or more grade (*p* = 0.335) in the riluzole group in comparison to the control group, though not to a level of statistical significance. On *post hoc* multi-variate linear regression, a statistically significant improvement was observed in the AIS C group that received riluzole for total motor score (*p* = 0.011) and upper motor score (*p* = 0.016) at 6 months post-injury in comparison to controls. Statistically significant improvements were also observed for functional outcomes regarding SCIM III total score (*p* = 0.036), SCIM III subgroups for self-care (*p* = 0.032), and respiratory management (*p* = 0.041), as well as the mental component score of Short Form Health Survey (SF36; *p* = 0.012) and EQ5D health state (*p* = 0.031).

SCIM III outcomes were also investigated in smaller patient populations by Kumarasamy and colleagues with a total of 43 patients between the two study groups, which also showed improvement in SCIM III outcomes in the riluzole group in comparison to the control group, though not to a level of statistical significance at 6 weeks (*p* = 0.63), 3 months (*p* = 0.99), 6 months (*p* = 0.95), or 1 year (*p* = 0.68).^22^ Kumarasamy and colleagues was also the only study that evaluated ASIA sensory outcome and showed an improvement in patients who received riluzole in comparison to control, though not to a level of statistical significance at 6 weeks (*p* = 0.41), 3 months (*p* = 0.21), 6 months (*p* = 0.32), or 1 year (*p* = 0.39).^22^

In addition to riluzole's mechanism as an antiepileptic medication, it has some reported anesthetic effects.^35^ Two of the studies included for meta-analyses investigated the Numerical Pain Rating Scale (NPRS) at 3 and 6 months post-SCI and was extended out to 1 year by Kumarasamy and colleagues.^22,26^ Kumarasamy observed a decrease in NPRS in the riluzole group in comparison to control, but not quite to a level that was statistically significant: 3 months (*p* = 0.76); 6 months (*p* = 0.43); and 1 year (*p* = 0.06).^23^ A statistically significant decrease in NPRS was observed by Meshkini and colleagues in the riluzole group in comparison to the control, which observed a significant decrease in NPRS at the 6-month follow-up and a nearly significant decrease at the 3-month follow-up: 3 months (*p =* 0.053); 6 months (*p* = 0.001).^26^

Although the focus of this article was on the safety and efficacy of riluzole for traumatic SCI in the acute setting, there may also be some utility in the chronic setting. In the chronic setting of SCI, riluzole has been observed in pre-clinical rat models to reduce spasticity.^36^ Similar effects have been observed in human patients with chronic SCI.^37^ There is currently a phase 1b-2b clinical trial investigating the safety and efficacy as it relates to spasticity in patients with chronic traumatic SCI.^38^

Overall, the results of this study suggest that riluzole may be a safe pharmacological treatment option in the setting of acute traumatic SCI. However, the results of our pooled meta-analyses showed marginal benefit in neurological outcomes. These results should prompt further study into the efficacy of riluzole. Large, randomized trials should be performed to establish the benefit of riluzole compared to placebo for the management of traumatic SCI. Specific attention should be given to parse out the ideal dose, duration, and time frame for administration, as well as subanalyses to determine which patient populations yield the most significant improvement.

### Limitations

This study is limited by the pooled data available from the relatively small series included in the analyses. Additionally, there was no standardized dose or duration of riluzole among the included studies, nor was there a standardized control intervention for comparison groups. In regard to demographics, some of the studies were represented by a high male-to-female ratio, even after accounting for a male predilection for traumatic SCI in males, which is typically 3 to 4 times higher than females.^39^

## Conclusion

The results of these meta-analyses suggest that riluzole for the treatment of traumatic SCI results in improved neurological outcomes with respect to ASIA motor score at 3 and 6 months post-injury, as well as change in AIS at 3 and 6 months, when compared to control, though not to a level of statistical significance. No specified adverse events related to riluzole were observed and riluzole resulted in fewer adverse events and lower mortality in comparison to control, though not to a level of statistical significance. More robust prospective randomized studies are necessary to help inform the safety and efficacy of riluzole for traumatic SCI.

## Abbreviations Used

AIS: ASIA Impairment Scale
ALS: amyotrophic lateral sclerosis
ALT: alanine aminotransferase
ASIA: American Spinal Injury Association
AST: aspartate aminotransferase
BID: twice-daily
CI: confidence interval
FDA: U.S. Food and Drug Administration
GI: gastrointestinal
GU: genitourinary
MD: mean difference
MP: methylprednisolone
NPRS: Numerical Pain Rating Scale
OR: odds ratio
SCI: spinal cord injury
SCIM: Spinal Cord Independence Measure
